# Can the primary health care model affect the determinants of neonatal, post-neonatal and maternal mortality? A study from Brazil

**DOI:** 10.1186/s12913-019-3953-0

**Published:** 2019-02-26

**Authors:** Alexandre Bergo Guerra, Luciane Miranda Guerra, Livia Fernandes Probst, Brunna Verna Castro Gondinho, Gláucia Maria Bovi Ambrosano, Estêvão Azevedo Melo, Valéria Silva Cândido Brizon, Jaqueline Vilela Bulgareli, Karine Laura Cortellazzi, Antonio Carlos Pereira

**Affiliations:** Department of Community Dentistry – Piracicaba Dental School, UNICAMP, Caixa postal 52, 13414-903, Piracicaba, São Paulo Brazil

**Keywords:** Infant mortality, Maternal mortality, Socioeconomic factors, Family health, Primary health care

## Abstract

**Background:**

The state of São Paulo recorded a significant reduction in infant mortality from 1990 to 2013, but the desired reduction in maternal mortality was not achieved. Knowledge of the factors with impact on these indicators would be of help in formulating public policies. The aims of this study were to evaluate the relations between socioeconomic and demographic factors, health care model and both infant mortality (considering the neonatal and post-neonatal dimensions) and maternal mortality in the state of São Paulo, Brazil.

**Methods:**

In this ecological study, data from national official open sources were used to conduct a population-based study. The units analyzed were 645 municipalities in the state of São Paulo, Brazil. For each municipality, the infant mortality (in both neonatal and post-neonatal dimensions) and maternal mortality rates were calculated for every 1000 live births, referring to 2013. Subsequently, the association between these rates, socioeconomic variables, demographic models and the primary care organization model in the municipality were verified. For statistical analysis, we used the zero-inflated negative binomial model. Gross analysis was performed and then multiple regression models were estimated. For associations, we adopted “p” at 5%.

**Results:**

The increase in the HDI of the city and proportion of Family Health Care Strategy implemented were significantly associated with the reduction in both infant mortality (neonatal + post-neonatal) and maternal mortality rates. In turn, the increase in birth and caesarean delivery rates were associated with the increase in infant and maternal mortality rates.

**Conclusions:**

It was concluded that the Family Health Care Strategy was a Primary Care organization model that contributed to the reduction in infant (neonatal + post-neonatal) and maternal mortality rates, and so did actors such as HDI and cesarean section. Thus, public health managers should prefer this model when planning the organization of Primary Care services for the population.

## Background

Maternal and infant mortality are serious public health events and the majority of these are readily preventable. Classically, this has allowed them to be considered the best indicators of the standard of living and social well-being of a population [1]. Their rates are presented as the annual number of female deaths from any cause related to or aggravated by pregnancy or its management during pregnancy and childbirth or within 42 days of termination of pregnancy, respectively, expressed per 100,000 live births; and the probability of an infant dying between birth and age of 1 year per 1000 live births, respectively [2].

Infant mortality has two additional dimensions, the neonatal mortality rate, which is the probability of a child dying during the period from 0 to 27 days of life, expressed per 1000 live births, and the post neonatal period, in which a child dies within 28 days up to completing 1 year of age, expressed per 1000 live births. These dimensions are relevant, because the first month of life is the period in which the infant is most vulnerable and most susceptible to dying, from the point of view of health care organizations [2, 3].

Over the years, the World Health Organization (WHO) and the United Nations (UN) have coordinated global efforts to reduce these rates, which have declined dramatically throughout the world. However, the desired goals of the Millennium Development Goals were not met, because despite the reduction of approximately 45% in maternal mortality and over 50% in neonatal and infant mortality, these events occurred in heterogeneous ways, with smaller or even lower reductions in the most vulnerable populations [4].

To reduce these rates, the WHO recommends postnatal care in facilities for at least 24 h after birth, and postnatal contact within 24 h after birth for newborns born at home. Thus, interventions are focused on the delivery period and soon after birth, based on the cause of death, with some efforts to reduce neonatal deaths beyond the first week after birth [5].

In recent decades, the struggle against maternal and infant mortality has intensified in Brazil, with public policies and greater allocation of resources to reduce it. With the aim of coordinating various sectors for the purpose of improving the quality of life of women and children, in 2004, Brazil established the “Maternal and Neonatal Mortality Reduction Pact”. The principles of this Pact are as follows: respect for the human rights of women and children; consideration of gender issues, ethnic and racial aspects, and social and regional inequalities; the political decision to invest in improving obstetric and neonatal care, including wide mobilization and participation of social managers and organizations [6].

With these actions, it was possible to make significant progress in reducing infant mortality rate (IMR) to a rate of 14 deaths per thousand live births in 2013, since it anticipated Millennium Development Goal 4, which was 15.7 per thousand live births, in addition to representing a fall of 78% in this rate between 1990 and 2013 [3]. In turn, the state of São Paulo recorded an IMR of 31.2 in 1990, which dropped to 11.66 per one thousand in 2013 and 11.4 in 2014, with a significant reduction since 2010 [7]. However, when we analyzed the dimension of neonatal mortality, we found that the country maintained high levels, with a rate of 11.2 deaths per thousand live births in 2010. These mortality levels are below the levels the country is potentially capable of achieving, and can be considered reflections of the unfavorable conditions of life of population and health care, in addition to historical regional and socioeconomic inequalities [6, 8].

When considering maternal mortality (MMR), despite the reductions observed, the results are less encouraging. The situation in Brazil has improved, as a decline in maternal mortality was shown from 141 per 100,000 live births in 1990 to 68 per 100,000 live births in 2010, but the goal of reducing ¾ of the target between 1990 and 2015 has not yet been reached [9]. Between January and September 2011, the maternal mortality rate declined by 21% [6, 8]. In the first decade of the twenty-first century, in the State of São Paulo, MMR was not expected to achieve the Millennium Development Goal 5: “Improve Maternal Health by presenting MMR equal to or lower than 35 deaths per 100,000 live births by 2015”. This was because only a slight drop was observed in this period, considering that in 2000, there were 275 maternal deaths (40.0) and in 2011, there were 249 deaths (40.8) [10]. Considering the magnitude of this problem, little change was produced in maternal mortality relative to the prenatal period. Thus, there was a need for more knowledge about this relevant issue, with the aim of promoting the health of pregnant and postpartum women [11].

Over the last decade, several studies have proven that both IMR and MMR were mightily related to contextual variables. The American study showed that improvements in primary care expressively influenced IMR and the number of underweight children in the US, thus demonstrating a negative association between the implementation of primary health care and reduction in infant mortality in the USA [12]. However, few studies have discussed how political and economic contexts shape the effects of health and environment, so that a politically and economically unstable society would have difficulties with transferring health resources for effectively implementing care measures for the populations’ health, in spite of this society having a sufficient number of professionals and health facilities to meet these requirements [13]. Therefore, research has the important task of reducing maternal mortality and its multi-causal factors around the world.

Although the family health care strategy (FHS) has a significant concentration of professionals and health services because of the latest measures implemented, it may still present the typical weaknesses of its immaturity. Therefore, it is necessary to carefully evaluate the different factors that may have affected maternal and infant mortality in the state, including the time since implementation of the FHS. The authors believe that this is the only way that will make it possible to discriminate the factors and their impacts without bias, and thus, to focus efforts on those that can effectively lead to improvement in these indicators.

Therefore, this study aimed to evaluate the relations between socioeconomic, demographic factors, the health care model and infant mortality (considering both the neonatal and post-neonatal dimensions) and maternal mortality in the state of São Paulo.

## Methods

### Study design

This was an ecological study with cross-sectional analysis of data obtained in 2013 concerning the state of São Paulo, Brazil.

### Ethical aspects

This ecological study was submitted to a local Research Ethics Committee (REC), according to Resolution no 466/12 of December 12th, 2012, from the National Health Council and complementary resolutions (240/97, 251/97, 292/99, 303/2000, 304/2000, 340/2004, 346/2005 and 441/2011). However, as secondary data from public databases were used, this study was exempted from consideration by the REC, according to REC Office 009/2016.

### Outcomes measures and data sources

Creation of the database and categorization of the studied variables:

Individual spreadsheets and search for databases were initially created on the websites of the bodies responsible (described below) for each variable studied. After the search, the data were transported to an Excel spreadsheet through the PROCV command for database management. For the municipalities that did not have data provided by the above-mentioned responsible bodies, the endpoint symbol (.) was assigned in its box (method used to recognize the SAS program to generate the analysis).

The following were the definitions and categorization of the studied variables:

- Population: Described the total number of the population according to IBGE census or projections [14]. Data provided by the agency (raw data) were used.
- Gross Domestic Product per capita (PIB per capita): Gross data [14].
- Human Development Index (HDI value): The raw data obtained from the State System of Data Analysis Foundation (SEADE-SP) [7] were used. The Human Development Index (HDI) is a measure of the degree of economic development and the quality of life offered to the population of countries and that serves as a comparison between them. This was calculated based on economic and social data, ranging from 0 (no human development) to 1 (total human development). The closer to 1, the more developed the country is. In summary, this is a synthetic indicator of quality of life that - in a simplified way - adds and divides the income, health and education of a given population into three levels [15]. Although the state of São Paulo – according to the three levels of income - has the highest HDI of all Brazilian states (0.783), when this was evaluated at the municipal level, as in our study, there was strong heterogeneity among the 645 municipalities, ranging from municipalities with HDI (0.639) to municipalities with very high HDI (0.862).
- Family Health Strategy implemented: Number of FHS teams implemented, provided by DATASUS [16]. Since 1994, Family Health has been the priority strategy for reorienting the care model based on primary care, in accordance with the principles of the Unified Health System [15]. It is a way of working health with families and communities as the center of attention based on the assumptions of primary health care, scored by Starfield, which seeks to overcome the idea of simplification and low cost for this level of attention (first contact, longitudinally, coverage of care, coordination and guidance provided to families and communities) [17]. Despite being the richest state in Brazil, São Paulo showed a slower and more heterogeneous implementation of the family health care strategy.
- Time of implementation of the Family Health Strategy: The time was calculated by the sum of the years of the first implementation until 2013 (data collection date) and transformed into months. The same was done for the time of the last deployment. These values were calculated individually for each municipality. Data provided by DATASUS [16].
- Proportion of FHS: Data on the estimate of the proportion of the population covered by the FHS were obtained through the criterion of one team for each 3450 people (standard calculation adopted by the Department of Primary Care of the Ministry of Health). Data provided by DATASUS [16].
- Community Health Agent (ACS) Proportion: Data on the estimated proportion of the population covered were obtained using the ACS criterion for every 575 persons (standard calculation adopted by the Ministry of Health - Basic Attention Department). Data provided by DATASUS [16].
- Neonatal Mortality Rate: Data obtained by the Information System on Mortality (SIM) and collected by the Information System on Live Births (SINASC) DATASUS [16]. This was calculated by the number of deaths in the age range of 0 to 27 days per thousand live births.
- Post-neonatal mortality rate: Number of deaths in the age range from 28 days to less than 1 year per thousand live births. SIM and (SINASC) DATASUS [16].
- Infant Mortality Rate: corresponded to the number of children dying between birth and age of 1 year per 1000 live births. This represented the sum of the neonatal mortality rate and the post-neonatal mortality rate. Data were obtained by the Mortality Information System (SIM) and collected by the Live Birth Information System (SINASC) DATASUS [16].
- Maternal mortality rate: The calculation was made by the number of deaths of women, due to causes related to pregnancy, childbirth and puerperium divided by the number of live births of resident mothers * 1000. Data were provided by DATASUS [16]. Although the maternal mortality rate is traditionally represented by 100,000 live births, in the present study this rate was presented per 1000 live births. This adaptation was necessary so that the data could be interpreted more adequately by the decision makers. For example, of the 645 municipalities in São Paulo, 506 had a population of up to 50,000, and 267, a population of up to 10,000 inhabitants [14]. Thus, when we considered the municipality as our unit of analysis, the presentation of the mortality rate in its traditional version, per 100,000 live births, would not represent adequate practical information for management in public health.
- Birth rate: Calculation was made by the number of live births divided by the total population number * 1000. (SINASC). Data were provided by DATASUS [16].
- Cesarean delivery rate: Data were provided by TABNET / DATASUS [16]. Calculation was made by the number of cesarean deliveries divided by total number of deliveries (normal + cesarean births) * 100. Data were provided by DATASUS [16].
- Percentage of live births to mothers receiving no prenatal consultation: Calculation = % = (Number of live births to women without consultation / number of live births) * 100. Data were provided by DATASUS [16].
- Vaccination Targets: total number of vaccinations for coverage of children under one year of age - was assigned to those municipalities that had vaccine coverage equal to or greater than 95%. Data provided by DATASUS [16].

After all the adjustments and checking data generated in the worksheets, these were sent to the statistician responsible for performing the analysis and creating the tables.

### Statistical analysis

Association between the predictor variables and the outcomes was assessed using zero-inflated negative binomial regression models. The models were evaluated according to the deviance / degree of freedom and the Correct Akaike Information Criterion (AICC). Initially, individual models were set for each variable (unadjusted analysis). Variables with *p* < 0.20 in the individual analysis were tested in the multiple regression model (*p* ≤ 0.05). All analyses were carried out in the SAS statistical program [18].

## Results

Descriptive analysis of the variables showed the values of mean, median, standard deviation, maximum, and minimum, as in Table 1.

**Table 1:** General characteristics of the cities of São Paulo in 2013 (*n* = 645)

Variable	Mean	Std Dev	Median	Minimum	Maximum
IMR^a^ Neo	7.79	9.98	6.37	0.00	142.86
IMR Post neo^b^	3.87	7.17	1.44	0.00	71.43
MMR^c^	0.44	1.75	0.00	0.00	20.41
IMR (neo + post)^d^	11.83	13.66	10.21	0.00	214.29
Population	65,588.67	462,445.98	12,687.00	807.00	11,446,275.00
GDP^e^	22,624.60	18,527.99	18,054.66	7232.60	283,589.47
HDI^f^ (Value)	0.74	0.03	0.74	0.64	0.86
FHS^g^ (Implanted)	5.72	37.86	2.00	0.00	938.00
Starting time FHS^g^	115.40	67.83	148.00	0.00	189.00
Latest time FHS^g^	42.88	52.73	17.00	0.00	186.00
FHS^g^ proportion	55.42	39.40	61.37	0.00	100.00
ACS^h^ proportion	62.92	38.47	75.93	0.00	100.00
Natality	13.39	10.57	12.72	0.97	223.71
Caesarean rate	69.56	14.57	70.59	26.09	100.00
Living child	1.23	1.60	0.82	0.00	11.76
Vaccination coverage	74.78	19.78	72.22	17.75	257.50

^a^*IMR Neo* Infant Mortality Rate Neonatal - Number of deaths in the age of 0 to 27 days per thousand live births

^b^IMR Post neo- Mortality rate Post neonatal: Number of deaths in the age of 28 days to less than 1 year per thousand live births

^c^*MMR* Maternal mortality rate: number of deaths of women, due to pregnancy, childbirth and puerperium, number of live births of resident mothers times 1000

^d^IMR (neo + post) ^4^ - This represented the sum of the neonatal mortality rate and the post-neonatal mortality rate

^e^*GDP* Gross domestic product

^f^*HDI* Human development index

^g^*FHS* Family Health Care Strategy Estimates of the proportion of the population covered by the FHS were obtained through the criterion of one team for every 3450 people

^h^*ACS* Community Health Agent - Data on the estimate of the proportion of the population covered were obtained by the criterion of one ACS for every 575 persons

Table 2 shows individual and adjusted regression analyses for infant mortality rate (neonatal + post-neonatal). With the increase in HDI of the city, starting time of FHS implementation, time of the last implementation of FHS and proportion of FHS implementation, the infant mortality rate decreased. As the birth rate and caesarean delivery rate increased, the infant mortality rate also increased.

**Table 2:** Individual and adjusted regression analyses by the zero-inflated negative binomial model for the dependent variable infant mortality rate (neonatal + post-neonatal)

Variable	Individual analysis			Adjusted analysis		
	Estimate	^a^IC95%	*p*-value	Estimate	^a^IC95%	*p*-value
Population	−0.00	− 0.00; 0.00	0.4629			
GDP^b^ (per capita)	−0.00	− 0.00; 0.00	0.0253			
HDI^c^	− 11.46	−16.72; − 6.19	< 0.0001	−10.13	−17.06; − 3.21	0.0041
Starting time of FHS^d^	− 0.00	− 0.01; − 0.00	0.0005	− 0.00	− 0.02; − 0.01	< 0.0001
Latest time of FHS^d^	− 0.01	−0.01; − 0.00	0.0001	− 0.01	− 0.01; − 0.00	0.0001
FHS^d^ Proportion	−0.01	− 0.01; − 0.00	0.0010	−0.02	− 0.03; − 0.01	< 0.0001
ACS^e^ proportion	−0.01	− 0.02; − 0.00	0.0004			
Birth rate	0.23	0.15; 0.30	< 0.0001	0.17	0.09; 0.25	< 0.0001
Caesarean rate	0.02	0.01; 0.04	0.0005	0.02	0.01; 0.04	0.0055
Live births without prenatal	0.14	−0.01; 0.29	0.0766			

^a^Wald 95% Confidence Limits

^b^*GDP* Gross Domestic Product

^c^*HDI* Human Development Index

^d^*FHS* Family Health Care Strategy

^e^*ACS* Community Health Agent

Table 3 shows individual and adjusted regression analyses for the neonatal mortality rate. With the increase in HDI of the city, starting time of FHS implementation, time of the last implementation of FHS and proportion of FHS implementation, the neonatal mortality rate decreased. As the birth rate and caesarean delivery rate increased, the neonatal mortality rate also increased.

**Table 3:** Individual and adjusted regression analysis by the zero-inflated negative binomial model for the dependent variable neonatal mortality rate

Variable	Individual analyses			Adjusted analyses		
	Estimate	^a^IC95%	*p*-value	Estimate	^a^IC95%	*p*-value
Population	−0.00	− 0.00; 0.00	0.4629			
GDP^b^ per capita	−0.00	− 0.00; 0.00	0.0096			
HDI^c^	− 14.37	−19.40; − 9.34	< 0.0001	−10.09	−16.61; − 3.56	0.0024
Starting time of FHS^d^	− 0.00	−0.01; − 0.00	0.0007	− 0.02	− 0.02; − 0.01	< 0.0001
Latest time of FHS^d^	− 0.01	−0.01; 0.00	< 0.0001	− 0.01	−0.01; 0.00	0.0007
FHS^d^ Proportion	−0.01	− 0.02; 0.01	< 0.0001	− 0.02	−0.03; − 0.01	< 0.0001
ACS^e^ proportion	−0.01	− 0.02; 0.01	< 0.0001			
Birth rate	0.27	0.20; 0.35	< 0.0001	0.20	0.12; 0.3	< 0.0001
Caesarean rate	0.00	0.01; 0.04	< 0.0001	0.02	0.01; 0.03	0.0003
Live births without prenatal	0.03	−0.08; 0.14	0.6020			

^a^Wald 95% Confidence Limits

^b^*GDP* Gross Domestic Product

^c^*HDI* Human Development Index

^d^*FHS* Family Health Care Strategy

^e^*ACS* Community Health Agent

Table 4 shows individual and adjusted regression analyses for the post-neonatal mortality rate. With the increase in the HDI of the city, starting time of FHS implementation, time of the last implementation of FHS and proportion of FHS implementation, the post-neonatal mortality rate decreased. As the birth rate increased, the post-neonatal mortality rate also increased.

**Table 4:** Individual and adjusted regression analysis by the zero-inflated negative binomial model for the dependent variable post-neonatal mortality rate

Variable	Individual analysis			Adjusted analysis		
	Estimate	^a^IC95%	*p*-value	Estimate	^a^IC95%	*p*-value
Population	−0.00	−0.00; 0.00	0.4577			
GDP^b^ precipitate	−0.00	− 0.00; 0.00	0.0010			
HDI^c^	− 18.79	−24.30; − 13.28	< 0.0001	−10.70	− 16.36; − 5.03	< 0.0001
Starting time of FHS^d^	− 0.00	−0.01; − 0.00	0.0055	−0.001	− 0.02; − 0.01	< 0.0001
Latest time of FHS^d^	− 0.01	−0.14; − 0.01	< 0.0001	−0.01	− 0.01; − 0.00	0.0005
FHS^d^ proportion	−0.02	− 0.03; − 0.02	< 0.0001	−0.02	− 0.03; − 0.01	< 0.0001
ACS^e^ proportion	−0.05	− 0.09; − 0.02	0.0028			
Birth rate	0.26	0.18; 0.35	< 0.0001	0.20	0.11; 0.27	< 0.0001
Caesarean rate	0.02	0.00; 0.03	0.0075			
Live births without prenatal	0.05	−0.09; 0.20	0.4677			

^a^Wald 95% Confidence Limits

^b^*GDP* Gross Domestic Product

^c^*HDI* Human Development Index

^d^*FHS* Family Health Care Strategy

^e^*ACS* Community Health Agent

Table 5 shows individual and adjusted regression analyses for the maternal mortality rate. With the increase in the HDI of the city and proportion of FHS implementation, the maternal mortality rate decreased. With the increase in the caesarean rate, the maternal mortality rate increased.

**Table 5:** Individual and adjusted regression analysis by the zero-inflated negative binomial model for the dependent variable maternal mortality rate

Variable	Individual Analyses			Adjusted analysis		
	Estimate	^a^IC95%	*p*-value	Estimate	^a^IC95%	*p*-value
Population	−0.00	−0.00; 0.00	0.9297			
GDP^b^ precipitate	−0.00	− 0.00; 0.00	0.0413			
HDI^c^	− 29.03	−45.04; − 13.03	0.0004	− 30.7	−47.61; − 13.85	0.0004
Starting FHS^d^	− 0.00	−0.00; 0.00	0.5707			
Latest FHS^d^	− 0.01	−0.02; − 0.00	0.0229			
FHS^d^ proportion	−0.07	− 0.10; − 0.04	< 0.0001	−0.03	− 0.05; − 0.02	0.0002
ACS^e^ proportion	−0.06	− 0.10; − 0.03	< 0.0001			
Birth rate	0.01	−0.01; 0.03	0.4734			
Caesarean rate	0.19	−0.03; 0.41	0.0902	0.08	0.04; 0.12	0.0002
Live births without prenatal	1.32	−2.03; 4.67	0.4406			

^a^Wald 95% Confidence Limits

^b^*GDP* Gross Domestic Product

^c^*HDI* Human Development Index

^d^*FHS* Family Health Care Strategy

^e^*ACS* Community Health Agent

## Discussion

For the analysis of maternal and infant mortality in the state of São Paulo, our study considered the social and macroeconomic context and the variables related to the health care model. The state of São Paulo is economically privileged; however, it has not reached the Millennium Development Goal related to maternal mortality. In contrast, it reached the infant mortality rate target before the agreed period. Thus, the importance of in-depth study of the factors that affect these indicators is evident. It is fundamental to include variables linked to the health care model, as the state of São Paulo implemented the FHS in a slower and more heterogeneous way than most of the other states in the country, because this state already had a structured network of health services in 1994 (when implementation of the FHS began). Politically and technically, it was believed that even with federal incentives, it was not worth changing the model of care. This view has changed over the years.

Shi et al. verified that the effect of income inequality on infant mortality disappeared when the statistical analysis led to adjustment of the model for areas where there was an increase in primary health care coverage, especially in the regions of greater social inequality [12]. This emphasized the importance of an appropriate statistical model that took the model of care into account. In this study, we considered the variables related to the economic context and linked to the model of care, also including the time since implementation of the model (first and last implementation) to control the effect of the previously mentioned heterogeneity of FHS implementation in the state.

Optimizing infant health, by improving health monitoring, nutrition, vaccination schedules and living conditions, and implementing parental education programs to minimize unintentional injuries, may lead to reducing injuries to vulnerable children [19]. In addition, better socioeconomic and demographic conditions, and the development of population health seemed to have caused the decrease in the maternal mortality among African descendants, where higher gross domestic product (GDP) was associated with lower MMR [20]. While in 2014, Lourenço et al. [21] found that as there was growth in the GDP from 1998 to 2008, infant mortality decreased in the state of São Paulo, Brazil.

In another study [22], the WHO verified that almost all maternal deaths occurred in developing countries (99%). These countries have major public health problems. They struggle against poverty and suffer from limited access to health services, including antenatal care and childbirth. Optimizing child health by improving health monitoring, nutrition, vaccination schedules and living conditions, and implementing parental education programs to minimize unintentional injuries could reduce injuries in vulnerable children [19].

In Africa, the results of Dersarkissian et al., in 2013 demonstrated that better socioeconomic and demographic conditions, as well as population health development, appeared to cause a decrease in African maternal mortality, where a higher gross domestic product (GDP) was associated the lower RMM [20]. In Brazil, with the growth in GDP from 1998 to 2008, decreased infant mortality was found in the state of São Paulo, Brazil [21].

Although limited, evidence on community-based primary health care (CBPHC) has been shown to be indispensable for improving maternal, newborn and child health, and to provide guidance for reducing the still prevalent indicators of preventable maternal and infant mortality [23]. In Brazil, the state and national child nutrition programs adopted in recent decades have influenced the drop in infant and maternal mortality [24]. Other studies have provided convincing evidence that the measures for reduction of income disparity produced improvements in the health of children and mothers in a short time interval [25, 26]. Thus, the variable “model of care” always needs to be present in the studies on infant mortality, irrespective of the analysis made.

In 2014, Lourenço et al. [21] affirmed that there was a decrease in infant mortality in the state of São Paulo from 1998 to 2008. However, the authors found no significant difference between 2004 and 2008, and no difference in the non-continuity of impact of the care model on the drop in IMR, indicating the need for further studies to better investigate the issue of infant mortality after 2008.

In addition to analyzing a period subsequent to the one studied by the authors, as advocated by the cited study, the present research verified that data such as considering the deployment time of the care model, not only clarified the importance of the FHS in infant mortality, but also of its continuity. Our study confirmed the importance of the care model (in this case, the FHS) in the control of infant mortality when we found the significant association of IMR with both the time of starting implementation and time of the last implementation, and even with the proportion of FHS deployed. Furthermore, this study reaffirmed the importance of performing this model throughout the maternal and child cycle, as we found that this effect occurred not only on the general IMRs but also on their neonatal and post-neonatal components.

Another important finding of this study was the association of both neonatal and post-neonatal mortality rates with birth rates. Mendes et al. [27] in 2012, in a study conducted with the Brazilian population, affirmed that the drop in the birth rate, reduction in fertility and decrease in the population of children under one year old determined the greater care families took of their children and better health assistance provided by the Brazilian State. Moreover, according to the authors, pediatric care was very sensitive to social and epidemiological changes. This situation could also be verified in countries such as Morocco, where in spite of the actions and strategies adopted, persistent socioeconomic inequalities continued to hamper improvements in indicators of maternal and child health [27].

The association of neonatal infant mortality with cesarean delivery supported the statement that the lowest perinatal mortality rates corresponded to those of countries that have cesarean rates below 10%, according to the WHO [28], and this was related to the care model. However, according to Patah and Malik [28], analyses of cesarean section rates should be done in the light of the health care model in force and social and cultural characteristics of a given society. The model of obstetric care defined, doctor-patient relationship, economic incentives and use of medical technology were of extreme importance in achieving the cesarean delivery. Considering the possibility of a better doctor-patient relationship in the FHS model, based on continuity of care, it is expected that pregnant women assisted by the FHS will receive information and empowerment to at least take part in the decision about the type of delivery. This should decrease the chances of cesarean section if the patient has been properly informed about the risks and benefits of all types of delivery. In addition, the association here verified also reflected certain data related to the socioeconomic factor.

In regions where there was greater coverage by health plans, the rates of cesarean section were also higher [28]. This was because private physicians were usually paid by production and the cesarean section was better remunerated than vaginal birth and demanded less time to be performed, in addition to the possibility of performing the section by prior appointment. In 2013, the state of São Paulo had the largest coverage of private health insurance in the country (44.41%) [10]. Despite this, as far back as 2004, Dias and Deslandes [28] stated that even in public maternities, cesarean section rates were still higher than expected, because surgical indications were also governed by issues related to medical training and to cultural trends of assistance.

There are several complex issues related to medical training and culture, model of obstetric care and working processes that need to be reviewed so that these factors fit the national cesarean section rate, especially in the state of São Paulo, because other complex questions also affected the neonatal infant mortality rates.

Much has been done in the country, such as the greater appreciation of Primary Health Care by implementation and expansion of the FHS, insertion of women in the labor market, increased education and reduction in the inequality of income, but the reduction in MMR has taken place at a slower pace than was expected. In the state of São Paulo (HDI = 0.783) - the richest in the nation [10] - there was an even more intriguing situation because the MMR remained stable from 2000 to 2011, although the rate was much lower than the national rate in 2000. However, there was an approximation to the national rate in São Paulo, due to the continuous drop in the national rate and stagnation of the state rate.

Ruiz et al. [1] recommend that the HDI adjusted for inequality was the best predictor for IMR and MMR. Income inequalities in São Paulo and in Brazil, as measured by the Gini index, were 0.472 and 0.501, respectively. However, despite the numerical difference, evolution of these indices occurred at the same rate in São Paulo and in the country from 2004 to 2013. While Brazil went from a Gini index of 0.555 in 2004 to 0.501 in 2013, São Paulo went from 0.5239 to the present 0.472. This pointed out the fact that the better income conditions of classes C, D and E in Brazil in the last two decades, added to the fact that there were expanded and improved primary care networks of health services throughout the country may have contributed to the significant improvement in MMR. In São Paulo, where these improvements in income also appeared to have occurred, there was heterogeneity and slowness in the implementation of the FHS, especially in the first decade of the 2000s. Therefore, this factor seemed to be decisive in explaining the slower drop in the indicator in a state with socioeconomic conditions such as those of São Paulo. Health surveillance – pillar of the FHS – was the feature that enabled the health teams to “capture” the pregnant women in the territory, who were still unattended, thus providing them with access to prenatal care the timeliest possible way, thereby overcoming an important factor of impact on maternal and child mortality - the lack of access or unqualified access (with insufficient visits) to prenatal care.

As regards the care model, needed reduction in cesarean section rates in Brazil, and in this case, in the state of São Paulo, the need to review the medical education in Brazil is highlighted. This is still rooted in the biomedical hegemonic model, hospitalization and dependence on hard technologies. The family health model requires practices that focus on interdisciplinary, surveillance and teamwork actions. The reduction in cesarean sections is a topic that needs to undergo reformulation of the curricula of medical schools to emphasize the humanization of childbirth, importance of pregnant women’s autonomy for making the decision about the type of delivery, and especially empowerment of the physician to make a decision jointly with the pregnant woman, so that their decisions take precedence over financial or operational and administrative questions.

## Limitations of the study and suggestions for future researches

The major limitation of this study was that the relationship between the factor of exposure and the event might not occur at an individual level because it was an ecological study. However, the analysis made it possible to identify the factors that deserved a more detailed investigation by means of other study designs. The fact that this was an ecological study with cross-sectional analysis was an outstanding limitation, as it hindered the establishment of causal links, however, there was a longitudinal linkage by previous data collection, such as the variable “time of implementation of the FHS” (since 1998).

Moreover, we suggest the continuity of this study through longitudinal researches to verify maternal health care from the perspective of cause and effect. However, there are two other suggestions: the first is focus on the group denominated “*Near Miss”*- composed of women who present potentially lethal complications during pregnancy, which translates into a group with better and more frequent sources of information in cases of maternal death [29, 30]. The study of this group should allow determination of the variables that are capable of better elucidating the difficulties in reducing this indicator. The second suggestion would be to check the impact of the “Stork Network” in maternal and infant indicators, since the purpose of this important healthcare network implemented by the Ministry of Health was to structure and organize the maternal and infant health care in the country. However, as it was implemented in 2011 and our study used data from 2013, this network was not analyzed here, as we understood that conclusions could be unfeasible, hasty or biased.

Another limitation requiring consideration is related to the Information Systems available in Brazil, which may contain database fragmentations, under-registration or even methodological inconsistencies. In the poorer municipalities, failures in the recording systems are known to be even greater [31, 32]. The major challenge concerns the inadequate completion of the Declarations of Live Births and Deaths (DN and DO), although there have been undeniable improvements in registration systems in the first decade of the century and modification of the declaration models in 2011. This has led to more efficient registration of variables such as, for example, prenatal care [33]. However, in relation to SIM, the study by Maia et al. found high percentages of incompleteness, and the only variable component of the statement that obtained “optimal” classification was the gender of the child [34]. Other studies at national level have also highlighted the high percentage of incompleteness in SIM as regards the duration gestation, an important predictor of infant death [34, 35]. These deficiencies in filling out important variables have led to limitations in the potential use of the system for epidemiological studies [36]. However, although these limitations exist, it is important to note their frequency is low and they would certainly not change the conclusions of this study.

## Conclusion

The proportion of FHS coverage and HDI were factors that influenced the IMR and MMR outcomes, demonstrating the importance of investment in human development and in the FHS model in the state of São Paulo. In addition, neonatal infant mortality showed a correlation with the earliest and latest times of FHS implementation, and with birth rate and cesarean delivery factors, emphasizing the importance of not interrupting the FHS model and paradigmatic changes related to birth to decrease caesarean sections.

Recognizing factors associated with infant and maternal mortality allows the implementation of public policies in the social and health spheres, with specific focus on attenuating these factors and making it possible to optimize resources - already scarce - and thus enabling the longevity of such policies. For example, by increasing the value of financial transfers for natural births in SUS hospitals, this would certainly encourage an increase in natural birth procedures rather than performing cesarean delivery, which would certainly influence the reduction in maternal mortality, according to the present study. The continuation of compensatory policies - known to interfere positively in the improvement of living conditions, education and longevity of the population – will have an influence on the HDI.

## Abbreviations

ACS: Community health agents
DATASUS: SUS data computing department
FHS: Family health care strategy
GDP: Gross domestic product
HDI: Human development index
IBGE: Brazilian institute of geography and statistics
IMR: Infant mortality rate
MDGs: Millennium development doals
MMR: Maternal mortality ratio
REC: Research ethics committee
SIM: Mortality information system
SINASC: Information system on live births
UN: United Nations
WHO: World health organization
